# A Transoral Approach to Decompression Odontoidectomy With Posterior Wiring and Fusion for Pediatric Platybasia With Chiari Malformation

**DOI:** 10.7759/cureus.62754

**Published:** 2024-06-20

**Authors:** Nur Aidurra Zainudin, Mohd Hisam Muhamad Ariffin

**Affiliations:** 1 Orthopaedics and Traumatology, Hospital Universiti Kebangsaan Malaysia, Kuala Lumpur, MYS; 2 Spine Surgery, Universiti Kebangsaan Malaysia Medical Centre, Kuala Lumpur, MYS

**Keywords:** osteogenesis, invagination, craniovertebral, chiari, basilar, atlantoaxial

## Abstract

Basilar invagination in a Chiari malformation associated with osteogenesis imperfecta in the pediatric population is a rare entity. We report a case of a seven-year-old female who presented with sudden-onset bilateral spastic quadriplegia and evidence of a basilar invagination on MRI. She underwent emergency decompression of the impinging odontoid via transoral approach followed by posterior wiring and fusion of the C1 and C2 vertebrae. Imaging modalities such as dynamic CT and MRI play a major role in delineating any craniovertebral anomalies and neural impingement not easily identified in plain radiographs. Understanding the complex craniovertebral junction (CVJ) anatomy and the possible causes of such deformities is vital for ensuring proper diagnosis and management of these patients.

## Introduction

Chiari malformation is described as a basilar invagination of the caudal part of the cerebellum or medulla oblongata into the spinal canal due to the reduction of posterior cranial fossa volume leading to anterior compression [1]. It is often associated with craniovertebral junction (CVJ) anomalies, with the atlantoaxial instability causing the narrowing of the foramen magnum. Abnormal development of the CVJ can be seen on plain radiographs as bifid posterior arch of atlas, os odontoideum, odontoid retroflexion, occipitalized atlas, C2-3, C5-6 fusion, and syringomyelia. Imaging modalities such as dynamic CT scans and MRI play a major role in delineating any craniovertebral anomalies and neural impingement not easily detected in plain radiographs. MRI shows hindbrain herniation and a dynamic flexion-extension CT scan of a sagittal view can depict the instability. The treatment approach to subluxations involves anterior or posterior decompression with a posterior fusion in cases with evidence of instability while reducible basilar invagination may benefit from a single posterior approach involving decompression and fusion.

## Case presentation

**Figure 1:**
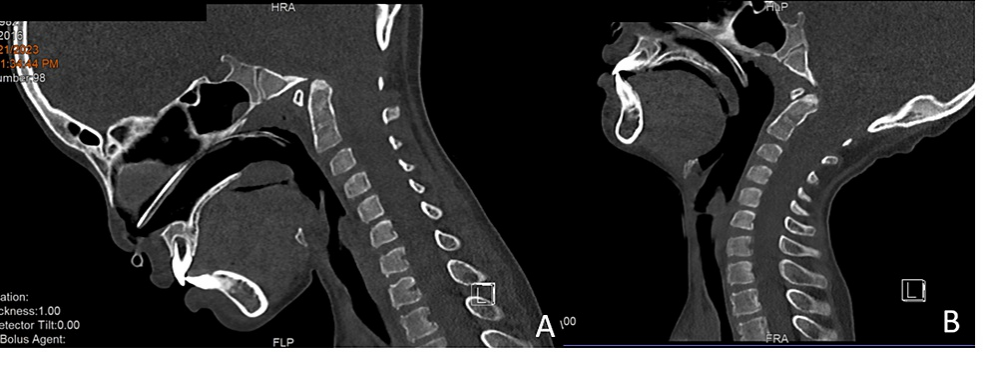
CT cervical in flexion (A) and extension (B) sagittal view on admission shows the widening of the spinal canal on extension CT: computed tomography

**Figure 2:**
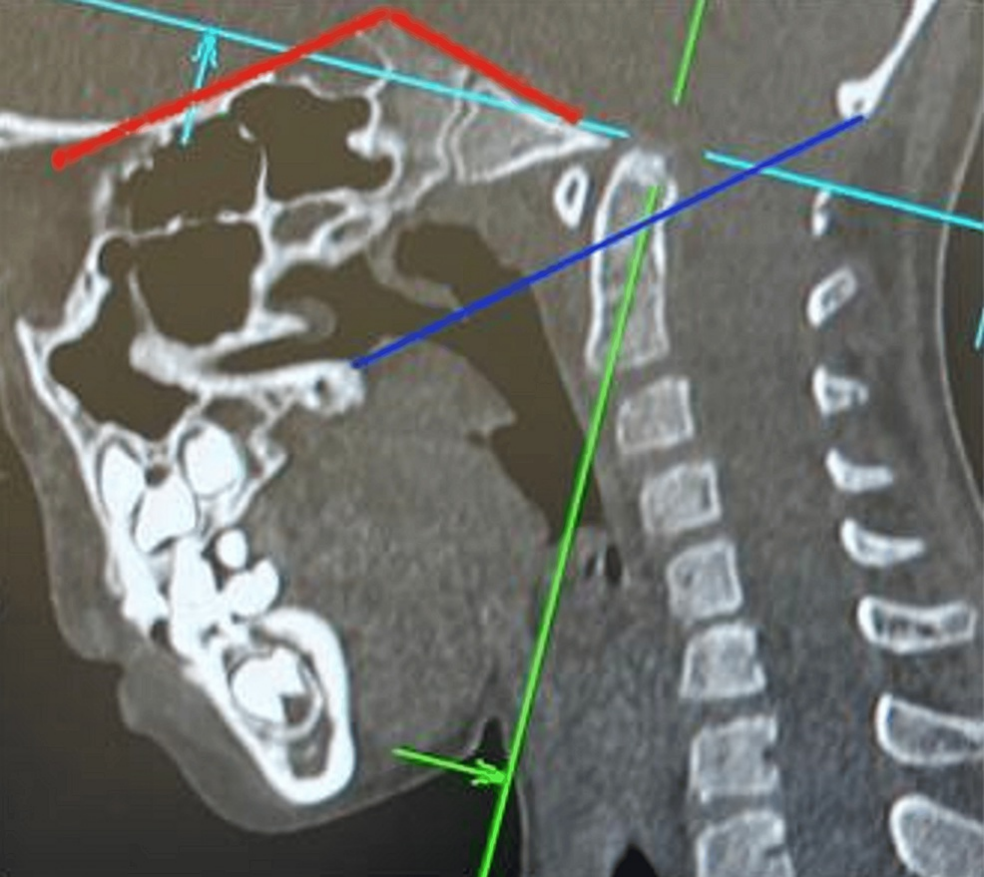
Craniometric lines and angles measured in the patient The Chamberlain line (blue line) joins the posterior margin of the hard palate to the opisthion. The tip of the dens is seen pressing into the brainstem. In normal patients, the tip of the dens should not exceed 5 mm above this line. Welcher basal angle (red line): the angle between the lines drawn from nasion to tuberculum sellae and tuberculum sellae to basion. The angle measured is 150 degrees, indicating a platybasia, as normal values range from 125 to 143 degrees

**Figure 3:**
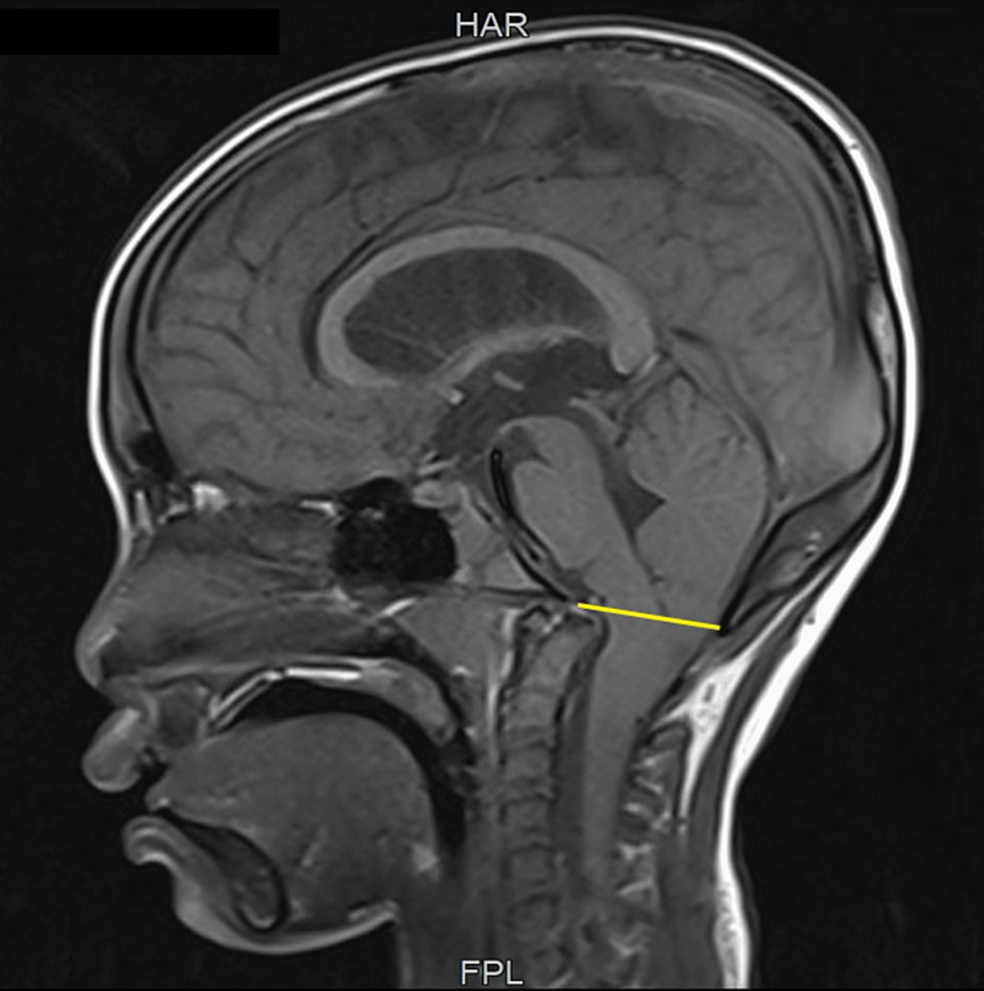
MRI showing the downward displacement of the cerebellum below the McRae line (yellow) indicating a Chiari malformation MRI: magnetic resonance imaging

The patient was a seven-year-old girl with normal birth and developmental history who was referred to us for spastic quadriplegia. She had a two-month history of frequent falls while playing with her siblings followed by the onset of bilateral lower limb weakness for two weeks before seeking treatment at a private hospital. She had initially been treated at the hospital for pneumonia with secondary bacterial infective meningitis. She had been intubated for 12 days and was noted to have an absent gag reflex. A general examination revealed features suggestive of osteogenesis imperfecta consisting of blue sclera, opalescent teeth, and a short stature. She presented with a decerebrate posture and spastic quadriplegia with a Medical Research Council (MRC) grade of power of 2. Neurological examination revealed hypertonic limbs, hyperreflexia, clonus, and a positive Babinski sign. We performed a dynamic CT scan of the cervical to visualize any atlantoaxial instability or CVJ defect and an MRI to assess the soft tissues involving neural structures, vascular supply, and surrounding ligaments. CT imaging showed basilar invagination of the cervical vertebra which improved on hyperextension of the neck (Figures 1-2). MRI showed downward displacement of the cerebellum below the McRae line and indentation of odontoid against the brainstem suggestive of a Chiari malformation with basilar impression (Figure 3).

**Figure 4:**
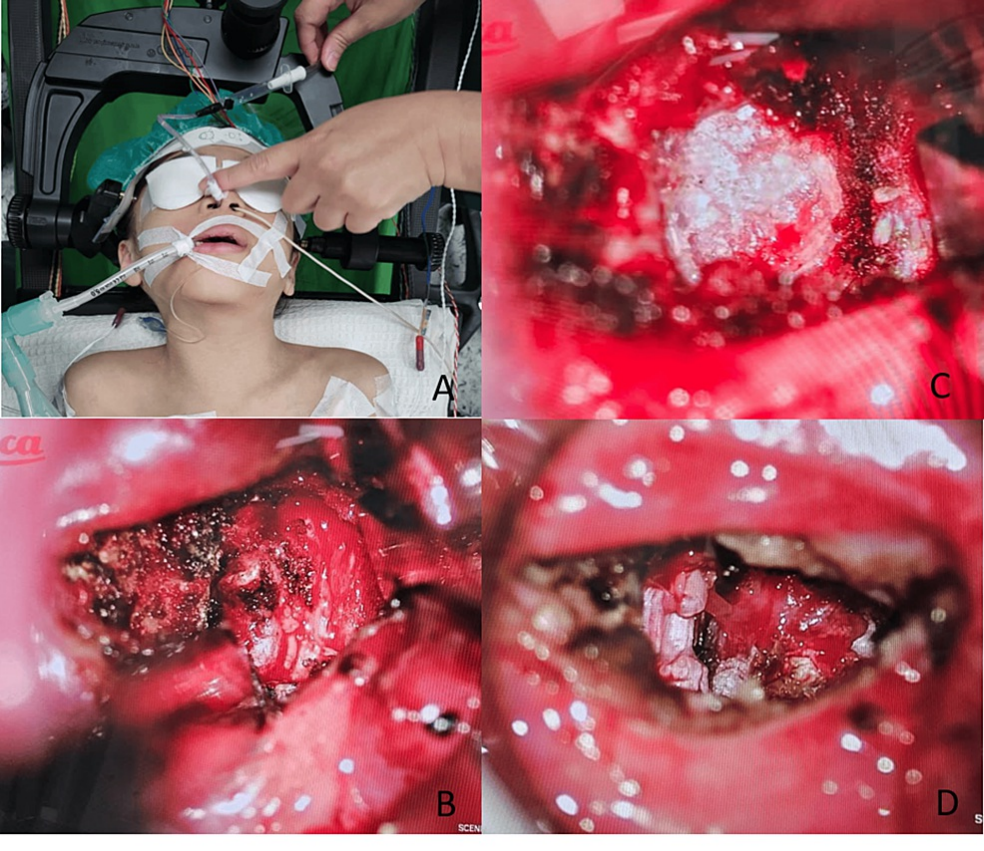
Surgery-related images of the patient (A) Patient position on Jackson operating table. (B) Visualized odontoid. (C) Remnant of odontoid after decompression. (D) Visualized transverse alar ligament

**Figure 5:**
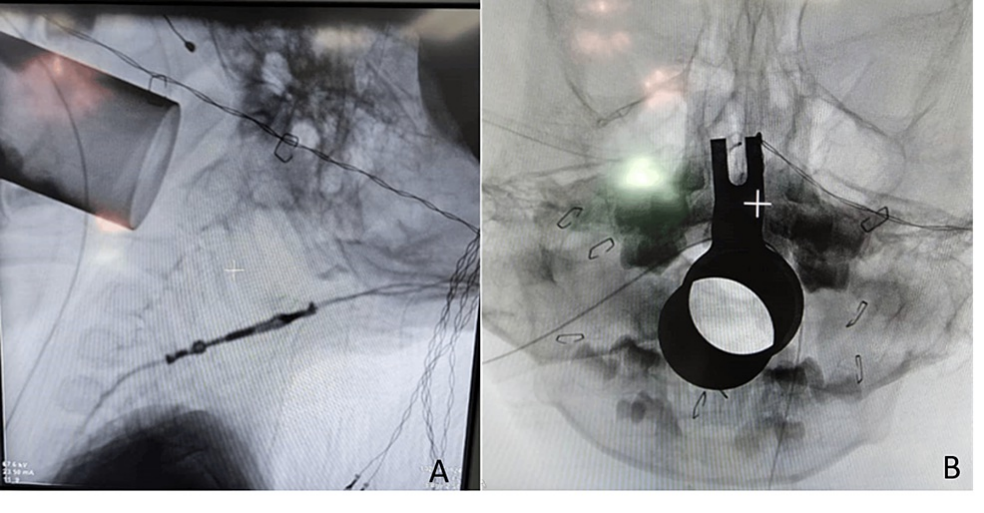
Image intensifier lateral and anteroposterior images of tubular retractor placement inside the oral cavity (A) The 90 mm tubular retractor placed directly onto the C1 anterior arch through the oral cavity in the lateral view of the image intensifier. (B) Placement of tubular retractor in anteroposterior view

She underwent a transoral resection of os odontoideum with C1-C2 arthrodesis via a dorsal wiring technique. During this surgery, the patient was positioned supine with her head inclined at 30 degrees (Figure 4). A 90 mm tubular retractor was inserted via the oral cavity and placed directly onto the C1 anterior arch. The position was then confirmed under image intensifier in lateral and anteroposterior views. The uvula and soft palate were pushed superiorly as it docked on the posterior pharyngeal wall (Figure 5). The surgical visualization field was further enhanced with the use of an operative microscope through the tube. A midline incision was made over the posterior pharyngeal wall and the layers were dissected to expose the anterior tubercle of C1. The os odontoideum was accessed by removing 1.5 cm of the anterior part of the C1 using a high-speed burr to decompress the spinal canal. Upon wound closure, the patient was repositioned prone in preparation for the posterior fusion of C1-C2 using cerclage wire with decortication of C1-C2 facet joint and packing with demineralized bone matrix.

**Figure 6:**
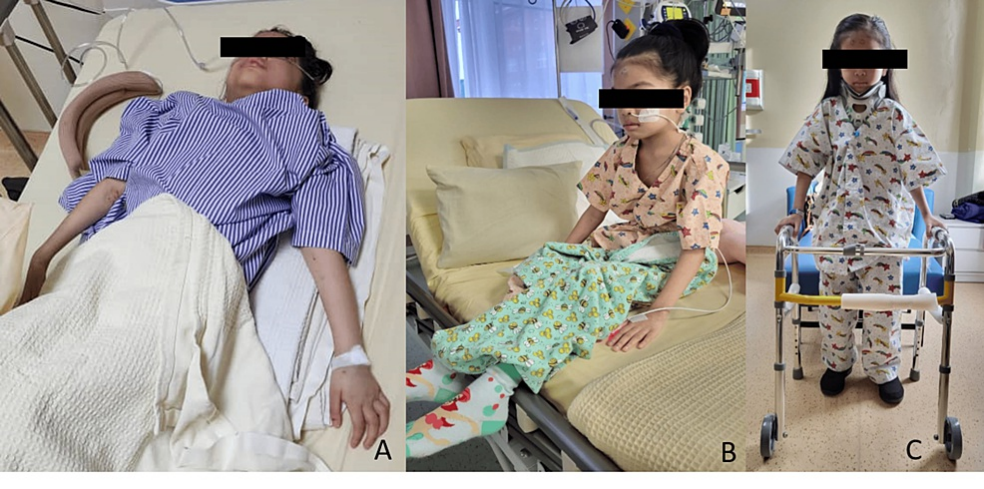
Patient images (A) On the first day of presentation. (B, C) Three weeks post-surgery

The patient was extubated the following day and underwent transoral odontoidectomy postoperative protocol. She was started on nasogastric tube feeding and maintained on a soft collar to assist with neck and head support. After three days post-surgery, she was readmitted to the ICU for aspiration pneumonia secondary to bulbar palsy causing severe oropharyngeal dysphagia and dysarthria. She had poor tongue movement, delayed swallowing, and weak cough reflex causing aspiration of her own saliva, which was evident by drooling of saliva, protruding tongue, and chesty cough. She underwent regular mucosal suctioning, chest physiotherapy, and nasogastric tube feeding until two weeks post-surgery, and she was then re-trained for oral feeding under the supervision of a speech therapist. Her neck and head control improved significantly after a week. She was started on intensive physiotherapy and rehabilitation to improve her upper and lower limb muscle strength alongside trunk and pelvic control. After three weeks of comprehensive rehabilitation, the patient was able to ambulate independently with a walker and was able to swallow solid food and speak in clear sentences (Figure 6).

**Figure 7:**
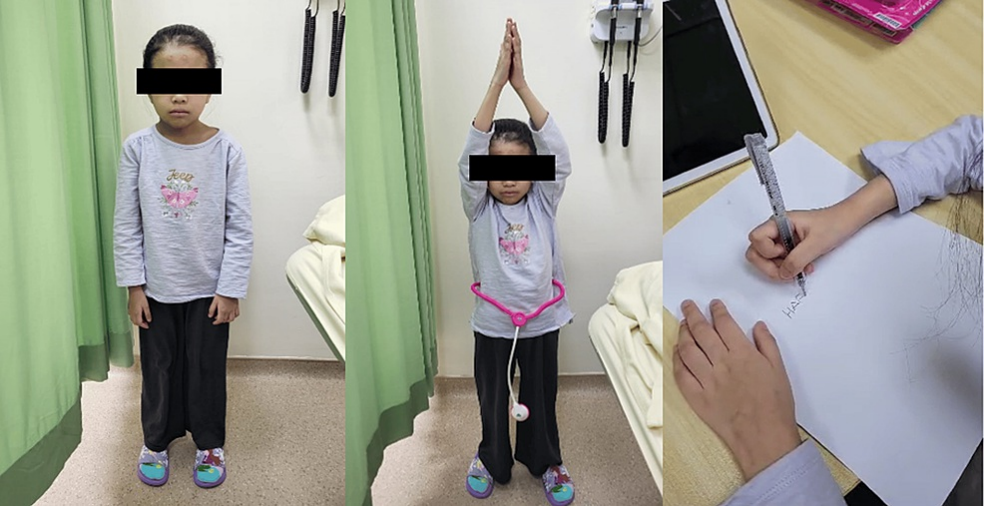
Patient at three months post-surgery

**Figure 8:**
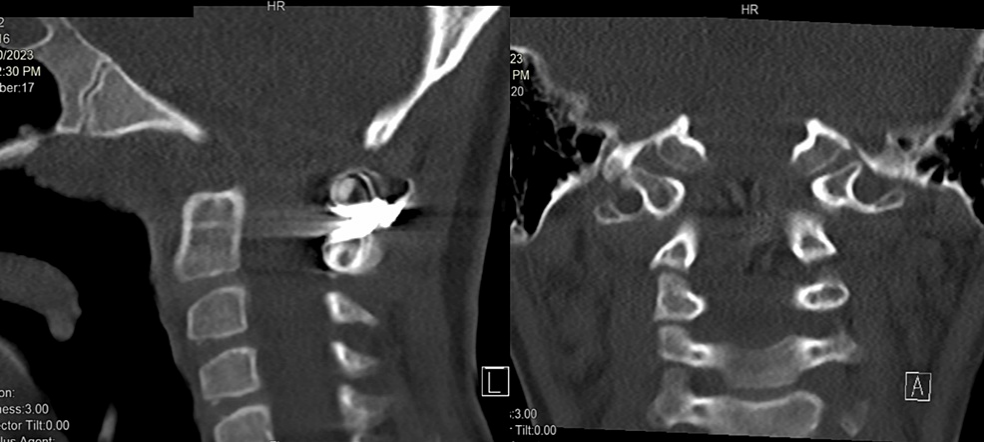
CT scan sagittal and coronal image at three months post-surgery shows a widening of the spinal canal CT: computed tomography

**Figure 9:**
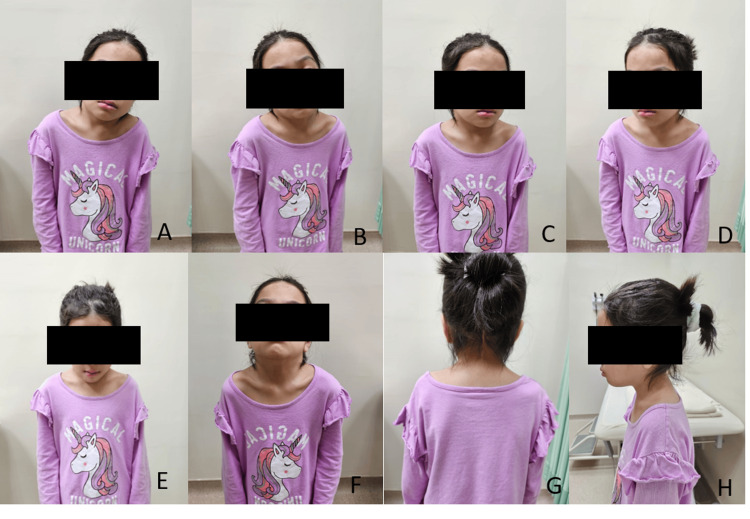
Patient’s neck range of motion at six months post-surgery (A, B) Neck range of motion in lateral flexion. (C, D) Lateral rotation. (E) Forward flexion. (F) Extension. (G, H) Neutral position

The patient was discharged in good condition and received regular clinic follow-up under our care to monitor her remarkable recovery. After three months post decompression, she was able to walk independently without any walking aids and has good fine motor movement, which includes handwriting, use of chopsticks, and pinching (Figure 7). She had good neck and truncal support and had an almost complete return to her normal condition before the surgery. A repeat CT scan of the neck revealed intact dorsal wires and a widening of the spinal canal (Figure 8). At six months post-surgery, the patient was ambulating normally, with full motor strength and intact sensation. There was some limitation in her neck range of motion, but it did not affect her daily activities such as wearing clothes, eating, writing, and playing with her siblings (Figure 9). A repeat X-ray showed intact dorsal wires and maintenance of the cervical alignment in the neutral position.

## Discussion

Craniovertebral congenital anomalies can manifest as a combination of multiple abnormalities involving bony and neural structures. It may result from an insult during intrauterine life causing failures of segmentation, fusion failure of different bone components, or hypoplasia and ankylosis [2]. A study by Menezes in 1995 found that 92 out of 100 patients with primary CVJ abnormalities and Chiari 1 malformation had common atlas assimilation with median basilar invagination. Of note, 66 patients presented with cervical C2-C3 vertebra segmentation defects, and 46 patients had syringohydromyelia [3].

Basilar invagination occurs when the odontoid abnormally prolapses into the foramen magnum and is attributed to congenital deformity of CVJ. It is commonly associated with clivus hypoplasia, occipital condyle hypoplasia, atlas hypoplasia, incomplete ring of C1 with lateral mass spreading, achondroplasia, and atlanto-occipital assimilation. Of note, 25-35% of cases reported central neurological involvement, which includes Chiari malformation, syringomyelia, syringobulbia, and hydrocephalus [4,5]. Basilar impression or platybasia is defined as an acquired form of basilar invagination caused by bone softening at the base of the skull and molding through the force of gravity. Clinically, it may present as shortness of the neck, and limitation of head movement with the head often carried at an unusual angle. These compounding deformities affecting the nervous system may present as paralysis and irritation of cranial nerves such as vagus and hypoglossal nerves, compression of cerebellum against tentorium, and local pressure effects upon medulla by the odontoid process and constricted foramen magnum [6].

Such cases of patients initially presenting with respiratory depression with subsequent neurological compromise due to basilar impression secondary to osteogenesis imperfecta were previously reported by Frank et al. in 1981 [7]. The patient was described as having neurological recovery after two months of undergoing posterior fossa and upper cervical decompression. Sawin et al. have described another similar case involving a boy with underlying osteogenesis imperfecta presenting with severe basilar invagination with indentation of the ventral brainstem by dysplastic clivus-odontoid complex. The patient underwent a transoral-transpalatopharyngeal resection of the distal clivus, anterior arch of the atlas, and odontoid process followed by a dorsal occipitocervical stabilization with titanium loop braided cables. His neurological symptoms resolved after six weeks post-surgery and he had a complete occipitocervical fusion at 18 months [8]. In our case, osteogenesis imperfecta was the likely predisposing factor for basilar invagination causing the patient's overall neurological deficit, as evidenced by the clinical manifestations of blue sclera, brownish opalescent teeth, and short stature.

## Conclusions

A thorough knowledge of the CVJ anatomy and its radiographic image interpretation is vital for deciding on the right treatment modality for these patients. Cases of acute paralysis causing respiratory distress require urgent decompression and fusion to relieve brainstem and cerebellar constriction. Good postoperative care involving a multidisciplinary team of physiotherapists, speech and language therapists, and rehabilitation physicians is essential to achieve patients' full recovery with good clinical and functional outcomes.
